# Implementation of “Treat‐all” at adult HIV care and treatment sites in the Global IeDEA Consortium: results from the Site Assessment Survey

**DOI:** 10.1002/jia2.25331

**Published:** 2019-07-12

**Authors:** Ellen Brazier, Fernanda Maruri, Stephany N Duda, Olga Tymejczyk, C William Wester, Geoffrey Somi, Jeremy Ross, Aimee Freeman, Morna Cornell, Armel Poda, Beverly S Musick, Fujie Zhang, Keri N Althoff, Catrina Mugglin, April D Kimmel, Marcel Yotebieng, Denis Nash, the IeDEA Consortium, Azar Karminia, Annette H. Sohn, Debbie Allen, Mark Bloch, Susan Boyd, Katherine Brown, Jess Costa, William Donohue, Manoji Gunathilake, Jennifer Hoy, Karen MacRae, Richard Moore, Norman Roth, Diane Rowling, Julie Silvers, . Smith, David Sowden, David Templeton, Rick Varma, Ian Woolley, David Youds, Somanithd Chhay Meng, Bun Vannary, Yun Ting Chan, Wilson Lam, Man Po Lee, Han Ning, Yu Po Chu Pansy, N. Kumarasamy, Sanjay Pujari, Nia Kurniati, Tuti Parwati Merati, Dina Muktiarti, Wayan Sandhi Parwata, Made Ratni, Ni Made Dewi Dian Sukmawati, Dian Sulistya Putu Diah Vedaswari, Ketut Dewi Kumara Wati, Evy Yunihastuty, Junko Tanuma, Graham Mills, Nigel Raymond, Rossana Ditangco, Ohnmar Seinn Papa, Ng Oon Tek, Ah‐neez, Raja Azwa, Dato, Fauziah Daud, Wong Ke Juin, Adeeba Binti Kamarulzaman, Nik Khairulddin, Chong Meng Li, Fong Siew Moy, Raja Iskandar Shah, Wong Peng Shyan, Benedict Sim, Jamal Mohamed Thahira, Koh Mia Tuang, Nik Yusoff, Jun Yong Choi, Yu‐Jiun Chan, Chih‐Sheng Huang, Wong Wing‐Wai, Anchalee Avihingsanon, Kulkanya Chokephaibulkit, Rawiwan Hansudewechakul, Benjhawan Khumcha, Suwimon Khusuwan, Sasisopin Kiertiburanakul, Pagakrong Lumbiganon, Alan Maleesatharn, Jutarat Praparattanapan, Thanyawee Puthanakit, Sirintip Sricharoenchai, Tavitiya Sudjaritruk, Suporn Watanaporn, Vu Thien An, Do Duy Cuong, Bùi Thu Hằng, Bùi Vũ Huy, Du Tuan Quy, Lam Nguyen Van, Agathomfue Baragunzwa, Dévote Gakima, Gloria Ingabire, Floride Kankinoi, Risase Scholastique Manyundo, Celestin Misago, Thierry Nahimana, Pélagie Nimbona, Felicite Ntirampeba, Christella Twizere, Rogers Ajeh, Amadou Djenabou, Anastase Dzudie, Alice Ndelle Ewanoge, Edmond Tchassem, Therese Bampapa, Patricia Lelo, Faustin Kitetele, Marie Paul, Amida Tytyna, Maryse Akolbout, Parfait Bitsindou, Merlin Diafouka, Adolphe Mafoua, Nadine Mahinga, Ella Moudila, Antoinette Moutoula, Ulrich Ndala, Dominique Mahambou Nsonde, Josephine Ayinkamiye, Chantal Dusabe, Theogene Hakizimana, Gilbert Mbaraga, Joyce Mukamana, Sandrine Mukantwali, Athanase Munyaneza, Anthere Murangwa, J. Claude Musenguwera, Marie Immanculee Ngutegure, Fidele Ntarambirwa, Diane Nyiransabimana, Jean d'Amour Sinayobye, Yvonne Tuyishimire, Olive Uwamahoro, Olive Uwamahoro, Habumuremyi Viateur, Sugira Vincent, Yee Yee Kuhn, Beverly Musick, Israel Rodriguez, Kara Wools‐Kaloustian, Constantin Yiannoutsos, Esinasi Akajoroit, Peter Ariya, Meshack Atsimale, Zeruya Barua, Oscar Busaka, Elizabeth Bukusi, Valentine Chebor, Timothy Chemweno, John Chirchir, Lameck Diero Sagida Esendi, Aisha Fwamba, Anne Mmella, Eunice Githumbi, Marcia Nasimiyu Hussein, Xavier Kandie, Martha Kemunto, Elizabeth Khaemba, Mary Kipchumba, Emily Koech, Caroline Kosgei, Barasa Laundrick, Ruth Merongo, Patricia Mochotto, Consolata Munyisi, Lilian Ndakalu, William Okoth Ochieng, Paul Odalo, Wicklife Okumu, Lilian Omari, Alphoce Omondi, Lydia Osia, Magret Owino, Maureen Oyoo, Doris Pepela, Millicent Rono, Omar Simon, Angie Tenge, Mary Too, Modesta Toto, Cathrine Towett, Kennedy Wawire, Mensaria Kimambo, Ester Kinyota, Rita Lyamuya, Julia Mathias, Athuman Ramadhan Mfuko, Denna Michael, Kapella Zacharia Ngonyani, Charles Nyaga, G.R. Somi, Mark S. Urassa, James Batte, Mwebesa Bosco Bwana, Barbara Castelnuovo, Michael Kanyesigye, Alice Kisakye, Fred Nalugoda, Haruna Semuwemba, John Ssali, Matthew Ssemakadde, Jessica Castilho, Carina Cesar, Paulo Ricardo de Alencastro, Eduardo Luiz Barbosa, Carlos Brites, Renata Caricol, Fabiana Bononi do Carmo, Lara Esteves Coelho, Maria Mercedes Escuder, Denize Lotufo Estevam, Flavia Gomes Faleiro Ferreira, Alexandre Gonçalves, Aída de Fátima Barbosa Gouvêa, Maria Leticia Rodrigues Ikeda, Artur O. Kalichman, Daisy Maria Machado, Simone Queiroz, Rosa de Alencar Souza, Regina Célia Succi, Kátia Valeska Trindade, Unai Tupinambás, Marcelo Wolff, Vanessa Rouzier, Denis Padgett, Brenda Crabtree, Carlos Eduardo Verne Martin, Fernando Mejia, Benny Chang, Brenda Done, Larry Gabe, John Gill, Kevin Gough, Gail Howlett, Marin Klein, Judy Latendre‐Paquette, Victor Leung, Paul MacPhee, P. MacPherson, Raj Maharaj, Lorna Carrasco Medina, Suzanne Page, Costas Pexos, Anita Rachlis, Kate Salters, Sherine Sterling, Stephen Boswell, Greer Burkholder, Gisela Cesteros, Kalliope Chagaris, Rosa Franklin, Jack Fuhrer, Cynthia L. Gilbert, Matthew Goetz, Chris Grasso, Michael Horberg, Robert F Hunter‐Mellado, Rita Kell, Mari Kitahata, Daniel Klein, Ken Levine, Vincent Marconi, Christopher Mathews, . Mayor, Catherine McGowan, Richard Moore, Sonia Napravnik, Richard Novak, Kris Ann Oursler, Shellier Ramos, Benigno Rodriguez, Maria C. Rodriguez, Michael Silverberg, Michael S. Simberkoff, Mohit Varshney, Douglas Ward, Barb Widick, Bienvenido G. Yangco, Mary‐Ann Davies, Lilian Smith, Per Maximilian von Groote, Josephine Muhairwe, Steve Balakasi, Quietus Banda, Getrude Kalepa, Bauman, Andrew Bello, J.W. Bulla, Maria Chigeda, Joyce Chikaphupha, Flora Chikwekwere, Jack Kachoka, Allan Kapito, Alinafe Nathan Katondo, Molly Kumwenda, Felix Phewa Labein, Ronald Magombo, Bridget Malumbe, I. Makuwira, Patricia Marico, Betha Masangale, Angella Mchiela, Dan Midian, Kezia Phiri, Mary Tambe, Baid Thomas, Charles Thomson, Jonas Hector, Anna Cross, Siphephelo Dlamini, Brian Eley, Jonathan Euvrard, Geoffrey Fatti, Katherine Hilderbrand, Marvin Hsiao, Michael Mpye, Hans Prozesky, Gary Reubenson, Lesley Rose, Shobna Sawry, Nosisa Sibambo, Karl Technau, Michael Vinikoor, Cleophas Chimbetete, Kamelia Kamenova, Eric Balestre, Valeriane Leroy, Karen Malasteste, Marcel Zannou Djimon, Marcelline D'Almeida, Ghislaine Hounhoui, Michee Assogba, Jacques Zoungrana, Issouf Yaméogo, Achille Tapsoba, Sidibé Abdelh, Clarisse Amani Bosse, Mamoudou Diabaté, Tanoh Kassi François Eboua, Madeleine Amorissani Folquet, Denise Hawelander, Mamadou Konaté, Kouadio Kouakou, Dohoun Lambert, Albert Kla Minga, Marie Sylvie N'gbeche, Aristophane Tanon, Abo Yao, Lorna Renner, Clémentine N'Diaye, Mme Alima Berthé, Moussa Seydi, Judicaël Tine, Takassi Ounoo Elom, Benjamin Kariylare, Akessiwe Patassi

**Affiliations:** ^1^ Institute for Implementation Science in Population Health City University of New York New York NY USA; ^2^ Graduate School of Public Health and Health Policy (GSPHHP) City University of New York New York NY USA; ^3^ Department of Medicine Division of Infectious Diseases Vanderbilt University Medical Center Nashville TN USA; ^4^ Department of Biomedical Informatics Vanderbilt University School of Medicine Nashville TN USA; ^5^ Vanderbilt Institute for Global Health (VIGH) Nashville TN USA; ^6^ National AIDS Control Programme Dar es Salaam Tanzania; ^7^ TREAT Asia, amfAR The Foundation for AIDS Research Bangkok Thailand; ^8^ Bloomberg School of Public Health Johns Hopkins University Baltimore MD USA; ^9^ School of Public Health and Family Medicine Faculty of Health Sciences University of Cape Town Cape Town South Africa; ^10^ Hôpital de Jour, Service des Maladies Infectieuses, CHU Souro Sanou Bobo‐Dioulasso Burkina Faso; ^11^ Institut Supérieur des Sciences de la Santé (INSSA) Université Nazi Boni Bobo‐Dioulasso Burkina Faso; ^12^ School of Medicine Indiana University Indianapolis IN USA; ^13^ Clinical and Research Center of Infectious Diseases Beijing Ditan Hospital Capital Medical University Beijing China; ^14^ Institute of Social and Preventive Medicine University of Bern Bern Switzerland; ^15^ School of Medicine Virginia Commonwealth University Richmond VA USA; ^16^ College of Public Health The Ohio State University Columbus OH USA

**Keywords:** HIV, “Treat all”, antiretroviral treatment, HIV care, guideline implementation

## Abstract

**Introduction:**

Since 2015, the World Health Organization (WHO) has recommended that all people living with HIV (PLHIV) initiate antiretroviral treatment (ART), irrespective of CD4+ count or clinical stage. National adoption of universal treatment has accelerated since WHO's 2015 “Treat All” recommendation; however, little is known about the translation of this guidance into practice. This study aimed to assess the status of Treat All implementation across regions, countries, and levels of the health care delivery system.

**Methods:**

Between June and December 2017, 201/221 (91%) adult HIV treatment sites that participate in the global IeDEA research consortium completed a survey on capacity and practices related to HIV care. Located in 41 countries across seven geographic regions, sites provided information on the status and timing of site‐level introduction of Treat All, as well as site‐level practices related to ART initiation.

**Results:**

Almost all sites (93%) reported that they had begun implementing Treat All, and there were no statistically significant differences in site‐level Treat All introduction by health facility type, urban/rural location, sector (public/private) or country income level. The median time between national policy adoption and site‐level introduction was one month. In countries where Treat All was not yet adopted in national guidelines, 69% of sites reported initiating all patients on ART, regardless of clinical criteria, and these sites had been implementing Treat All for a median period of seven months at the time of the survey. The majority of sites (77%) reported typically initiating patients on ART within 14 days of confirming diagnosis, with 60% to 62% of sites implementing Treat All in East, Southern and West Africa reporting same‐day ART initiation for most patients.

**Conclusions:**

By mid‐ to late‐2017, the Treat All strategy was the standard of care at almost all IeDEA sites, including rural, primary‐level health facilities in low‐resource settings. While further assessments of site‐level capacity to provide high‐quality HIV care under Treat All and to support sustained viral suppression after ART initiation are needed, the widespread introduction of Treat All at the service delivery level is a critical step towards global targets for ending the HIV epidemic as a public health threat.

## 1 Introduction

WHO's 2015 recommendation 1 for immediate treatment of all PLHIV, regardless of CD4+ cell count, represented a paradigm shift in HIV care and treatment. By preventing morbidity and mortality among PLHIV 2, 3 and averting new infections through onward transmission of the virus 4, 5, 6, universal treatment of HIV (also known as “Treat All” and “Test and Treat”) provides a clear strategy for ending the HIV epidemic and for meeting the targets set by the Joint United Nations Programme on HIV/AIDS in 2014 – that is, ensuring that 90% of PLHIV know their status, with 90% of those who are diagnosed (and therefore eligible) on combination ART, and 90% of those on ART achieving sustained viral load suppression by 2020, with 95‐95‐95% respectively, reaching these targets by 2030 7, 8.

**Figure 1:**
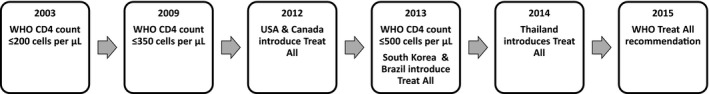
Timing of major HIV treatment guideline changes, including universal treatment of people living with HIV.

While evidence of the benefits of early treatment led a few countries to adopt Treat All in national policy guidelines prior to WHO's 2015 recommendation (Figure 1), translation of WHO guidance into national policies and into clinical practice at the service delivery level often lags, particularly in low‐resource settings. An analysis of 33 countries in sub‐Saharan Africa found that the time lag in national‐level adoption of WHO's 2009 and 2013 HIV treatment guidelines averaged 24 and 10 months, respectively 9. Other analyses of site‐level implementation of prior WHO guidelines have highlighted logistical challenges that contribute to delays in translating policies into practice 10, 11, 12. Although available evidence suggests that by late 2017, most countries around the world had adopted some form of the WHO's Treat All guidance 13, 14, little is known about the timing of site‐level introduction and how this varies across regions and levels of health care delivery, or about site‐level capacity to appropriately initiate all enrolled patients on ART.

Using data collected from health facilities that are part of the International epidemiology Databases to Evaluate AIDS (IeDEA) – a global collaboration that consolidates, curates and analyses longitudinal data on care and treatment of PLHIV – we sought to assess the status and timing of site‐level Treat All introduction for adult PLHIV across multiple regions and countries. We also aimed to describe site‐level practices related to pre‐ART counselling, the timing of ART initiation and viral load monitoring capacity at sites where Treat All is the standard of care.

## 2 Methods

### 2.1 Data sources

#### 2.1.1 Data collection

IeDEA is an international research consortium of HIV care and treatment sites in 46 countries across seven world regions: the Asia‐Pacific; the Caribbean, Central and South America; Central Africa; East Africa; Southern Africa; West Africa; and North America 15. IeDEA is a purely observational research consortium that does not dictate policies or practices to participating HIV care and treatment clinics. IeDEA regularly conducts general and specialized surveys in order to characterize the attributes, capacity and services available at sites that participate in the consortium 16, 17.

Between June and December 2017, a cross‐sectional 115‐item survey was administered in English or French to 255 active HIV care and treatment clinics that contribute longitudinal patient‐level data to IeDEA. In Southern Africa, where many IeDEA sites contribute data as part of a programmatic cohort that follows uniform practices across all clinics, one site from each cohort was surveyed; accordingly, four cohort‐representative sites were surveyed in Lesotho, Mozambique, Zambia and Zimbabwe, representing 8, 17, 105 and 35 clinics respectively, within each country's active observational cohorts.

The survey was distributed in paper form and as an online questionnaire. REDCap electronic data capture tools hosted at Vanderbilt University Medical Center 18 were used to implement the online version of the survey. Surveys completed on paper were entered into REDCap by regional representatives. All sites and IeDEA regional coordinating centres had IRB approvals in place permitting the collection of site‐level data for the survey.

#### 2.1.2 Site‐level characteristics and practices related to Treat All

The survey explored the current criteria used for initiating ART and the month and year those guidelines were introduced at the service delivery site. Adult treatment sites were considered as having implemented the Treat All policy if they reported that they currently “Start *all* patients on ART regardless of CD4+ cell count or symptoms.” Sites reporting that they only “Start *some* patients on ART regardless of CD4+ cell count or symptoms” – for example, patients with a pregnancy or coinfection with tuberculosis or hepatitis B – were not considered to be implementing Treat All.

Other survey items included questions related to facility attributes, including location (urban vs. rural), level (e.g. health centre, district hospital, regional/provincial and teaching hospital), and sector (public vs. private). The survey also explored routine site‐level practices related to pre‐ART counselling, ART initiation and viral load monitoring. Sites that reported that they could routinely request or perform viral load (quantitative HIV RNA) testing were considered to have the capacity for viral load monitoring among patients initiating ART.

#### 2.1.3 National HIV treatment guidelines and setting characteristics

To determine dates of national adoption of Treat All, we conducted a systematic search of health ministry websites for national HIV treatment guidelines, policies, notices, and press releases related to Treat All. When policy documents were unavailable or specified only the year of Treat All adoption, the date of policy adoption was assessed based on other sources, including the International Association of Providers of AIDS Care (IAPAC) Global HIV Policy Watch 19 and country operational plans (COPs) of the United States President's Emergency Plan for AIDS Relief (PEPFAR), as well as in‐country researchers and national treatment programme staff affiliated with IeDEA.

As national HIV treatment guideline changes were ongoing in 2017, we considered the national guideline to be that which was in place at the time each site completed the survey. Sites were considered to be subject to a national Treat All policy if they completed the survey after universal HIV treatment had been officially adopted in published national guidelines. Sites were considered to be operating within a pre‐Treat All policy context if they completed the survey before the date of national Treat All adoption.

We collected information on each country's income group classification in 2017 from World Bank databases 20 and status as a PEPFAR‐ and/or Global Fund to Fight AIDS, Tuberculosis and Malaria (Global Fund)‐supported country respectively, from the PEPFAR and Global Fund websites 21, 22. Countries with a PEPFAR country operational plan for 2017 were considered PEPFAR‐supported countries, and countries with a 2017 funding allocation were considered Global‐fund supported countries.

### 2.2 Statistical analysis

Analyses included descriptive statistics (frequency calculations, median year of site‐level Treat All implementation and median intervals – in months – between national guideline adoption and site‐level implementation). Frequencies of site‐level implementation of Treat All were stratified by site characteristics, country characteristics, and region, with Fisher's exact tests used to assess independence. The Wilcoxon test was used to compare medians. All statistical analyses were performed using SAS 9.4 (SAS Institute, Cary, NC), and ArcGIS Desktop 10.6 (Environmental Systems Research Institute, Redlands, CA) was used for descriptive mapping.

Sites that did not report the month of site‐level Treat All introduction were included in frequency calculations of the timing of Treat All introduction relative to (i.e. before/after) national guideline adoption if the year of site‐level introduction differed from the year of national guideline adoption. Sites were excluded if the year of site‐level introduction was the same as that of national adoption because there was no way to verify whether these sites began implementation before or after the national guideline adoption.

The interval between national guideline adoption and site‐level introduction was assessed as the time in months between the publication of updated national treatment guidelines that reflected Treat All and the month that each site reported beginning to provide ART to all HIV patients, regardless of CD4+ cell count or clinical disease staging. In countries where Treat All was not incorporated in national guidelines, we assessed the time, in months, between site‐level introduction of Treat All and the date the survey was completed as the duration of implementation. Sites that were unable to report both month and year of Treat All introduction were excluded from these analyses.

## 3 Results

Responses to the IeDEA Site Assessment survey were received from 234/255 IeDEA sites, including 201/221 sites (91%) in 41 countries that provide HIV care to adult patients. Thirty‐four sites providing services to paediatric patients only were excluded from the analysis because the timing of Treat All guidance for paediatric patients differed from that for adult patients.

### 3.1 Site‐level Treat All implementation

Overall, 93% of adult HIV treatment sites (187/201) reported that they currently initiate all patients on ART, irrespective of CD4+ cell count or WHO clinical stage (see Table 1). All IeDEA sites in the Caribbean, Central and South America and in East Africa regions reported implementing Treat All, as did the vast majority of sites in the Southern Africa, North America, Central Africa and Asia‐Pacific regions. In contrast, in the West Africa region – where national policies at the time of the survey did not reflect Treat All adoption in any country – 63% of sites reported that all patients were initiated on HIV treatment. There were no statistically significant differences in site‐level Treat All implementation by health facility type, urban/rural location, sector (public vs. private), country income group or PEPFAR/Global Fund‐support status.

**Table 1:** Implementation of Treat All at IeDEA sites, by national guideline status

	Participating sites			Sites implementing Treat All			Sites implementing Treat All prior to national guideline change^a^ (N = 170)
	All sites	In countries with national adoption of Treat All	In countries without national adoption of Treat All	All sites (N = 201)	In countries with national adoption of Treat All (N = 175)	In countries without national adoption of Treat All (N = 26)	
All sites	201 (100%)	175 (87.1%)	26 (12.9%)	187 (93.0%)	169 (96.6%)	18 (69.2%)	59 (34.7%)
IeDEA region [Fisher's exact test *p*‐value]				[*p* = 0.004]	[*p* = 0.446]	[*p* = 0.309]	[*p* < 0.0001]
Asia‐Pacific	42 (20.9%)	34 (81%)	8 (19%)	36 (85.7%)	31 (91.2%)	5 (62.5%)	21 (65.6%)
Caribbean, Central and South America	14 (7.0%)	10 (71.4%)	4 (28.6%)	14 (100%)	10 (100%)	4 (100%)	6 (50%)
Central Africa	19 (9.5%)	16 (84.2%)	3 (15.8%)	17 (89.5%)	16 (100%)	1 (33.3%)	3 (17.6%)
East Africa	42 (20.9%)	39 (92.9%)	3 (7.1%)	42 (100%)	39 (100%)	3 (100%)	13 (33.3%)
North America	41 (20.4%)	41 (100%)	0 (0%)	39 (95.1%)	39 (95.1%)	0 (0%)	11 (35.5%)
Southern Africa	35 (17.4%)	35 (100%)	0 (0%)	34 (97.1%)	34 (97.1%)	0 (0%)	0 (0%)
West Africa	8 (4.0%)	0 (0%)	8 (100%)	5 (62.5%)	0 (0%)	5 (62.5%)	5 (100%)
Health facility type				[*p* = 0.131]	[*p* = 0.363]	[*p* = 0.628]	[*p* = 0.011]
Primary (health centre)	101 (50.2%)	96 (95%)	5 (5%)	97 (96%)	94 (97.9%)	3 (60.0%)	22 (24.7%)
District hospital	18 (9.0%)	18 (100%)	0 (0%)	17 (94.4%)	17 (94.4%)	0 (0%)	6 (37.5%)
Regional/provincial or teaching hospital	82 (40.8%)	61 (74.4%)	21 (25.6%)	73 (89%)	58 (95.1%)	15 (71.4%)	31 (47.7%)
Sector				[*p* = 0.703]	[*p* = 1.00]	[*p* = 1.00]	[*p* = 0.129]
Public	169 (84.1%)	144 (85.2%)	25 (14.8%)	156 (92.3%)	139 (96.5%)	17 (68.0%)	45 (31.9%)
Private	32 (15.9%)	31 (96.9%)	1 (3.1%)	31 (96.9%)	30 (96.8%)	1 (100%)	14 (48.3%)
Facility location				[*p* = 0.121]	[*p* = 0.673]	[*p* = 1.00]	[*p* < 0.0001]
Urban/Mostly urban	149 (74.1%)	124 (83.2%)	25 (16.8%)	136 (91.3%)	119 (96.1%)	17 (68.0%)	54 (44.3%)
Rural/Mostly rural	52 (25.9%)	51 (98.1%)	1 (1.9%)	51 (98.1%)	50 (98.0%)	1 (100%)	5 (10.4%)
Country income group				[*p* = 0.751]	[*p* = 0.410]	[*p* = 0.453]	[*p* < 0.0001]
Low income	58 (28.9%)	50 (86.2%)	8 (13.8%)	54 (93.1%)	49 (98.0%)	5 (62.5%)	6 (11.1%)
Lower‐middle income	49 (24.4%)	37 (75.5%)	12 (24.5%)	44 (89.8%)	37 (100%)	7 (58.3%)	19 (47.5%)
Upper‐middle income	29 (14.4%)	27 (93.1%)	2 (6.9%)	28 (96.6%)	26 (96.3%)	2 (100%)	9 (34.6%)
High income	65 (32.3%)	61 (93.8%)	4 (6.2%)	61 (93.8%)	57 (93.4%)	4 (100%)	25 (50%)
PEPFAR‐supported country				[*p* = 0.093]	[*p* = 0.094]	[*p* = 0.683]	[*p* < 0.0001]
No	94 (46.8%)	80 (85.1%)	14 (14.9%)	84 (89.4%)	75 (93.8%)	9 (64.3%)	37 (52.1%)
Yes	107 (53.2%)	95 (88.8%)	12 (11.2%)	103 (96.3%)	94 (98.9%)	9 (75.0%)	22 (22.2%)
GFATM‐supported country				[*p* = 0.574]	[*p* = 0.231]	[*p* = 0.277]	[*p* < 0.0003]
No	76 (37.8%)	72 (94.7%)	4 (5.3%)	72 (94.7%)	68 (94.4%)	4 (100%)	30 (50.0%)
Yes	125 (62.2%)	103 (82.4%)	22 (17.6%)	115 (92.0%)	101 (98.1%)	14 (63.6%)	29 (26.4%)
Year of national Treat All adoption				[*p* < 0.0001]	[*p* = 0.060]	‐	[*p* < 0.0001]
2012 (2 countries)	41 (20.4%)	41 (100%)	‐	39 (95.1%)	39 (95.1%)	‐	11 (35.5%)
2013 (2 countries)	8 (4.0%)	8 (100%)	‐	8 (100%)	8 (100%)	‐	4 (50%)
2014 (2 countries)	6 (3.0%)	6 (100%)	‐	6 (100%)	6 (100%)	‐	1 (16.7%)
2015 (2 countries)	18 (9.0%)	18 (100%)	‐	16 (88.9%)	16 (88.9%)	‐	8 (61.5%)
2016 (16 countries)	97 (48.3%)	97 (100%)	‐	96 (99.0%)	96 (99.0%)	‐	15 (16.3%)
2017 (2 countries)	5 (2.5%)	5 (100%)	‐	4 (80.0%)	4 (80.0%)	‐	4 (100%)
Treat All not adopted nationally^b^ (15 countries)	26 (12.9%)	‐	26 (100%)	18 (69.2%)	0 (0%)	18 (100%)	16 (100%)

^a^Sites with known month and year of Treat All introduction; ^b^sites in countries that adopted Treat All in 2017 after the survey was completed counted among sites where Treat All was not yet adopted nationally.

In countries where universal treatment of PLHIV had been adopted in national treatment guidelines (26/41 countries), site‐level implementation of Treat All was almost universal (97%, 169/175 sites), and there were no significant differences in site‐level implementation by country income designation or PEPFAR‐support status, or by facility type, location or sector. Site‐level implementation of Treat All was significantly lower in countries where universal HIV treatment had not yet been incorporated into national guidelines (15/41 countries) at the time of the survey (69%, 18/26 sites), compared to countries where Treat All was adopted in national treatment guidelines (97%, 169/175 sites, *p* < 0.0001).

### 3.2 Timing of site‐level Treat All introduction

Among 178 sites that reported the year they began implementing Treat All, the year of site‐level introduction of Treat All ranged from 2008 to 2017 (Median year: 2016; IQR: 2015 to 2016) (Table 2). The median year of site‐level Treat All introduction was earliest in the North America (2014), Asia‐Pacific (2015), and Caribbean, Central and South America (2015) regions, and latest among sites in the West Africa region (2017). The median year of site‐level Treat All introduction was earlier at higher‐level health facilities (e.g. regional/provincial and teaching hospitals), private sector facilities, and sites located in high‐income, non‐PEPFAR‐supported countries.

**Table 2:** Timing and length of Treat All implementation at IeDEA sites

	Timing of Treat All implementation (sites with known year of implementation)		Time (in months) from national adoption to site implementation^a^ in countries where Treat All adopted nationally		Length of implementation (in months) among sites in countries where Treat All not yet adopted nationally	
	N	Median [IQR]	N	Median [IQR]	N	Median [IQR]
All sites	178	2016 [2015 to 2016]	138	1 [−1, 4]	14	7.4 [5.6, 12.7]
IeDEA region						
Asia‐Pacific	35	2015 [2014 to 2016]	24	−2 [−12, 5]	3	3.3 [0.6, 5.6]
Caribbean, Central and South America	13	2015 [2013 to 2016]	16	0 [0, 1]	2	10 [3.9, 16.1]
Central Africa	17	2016 [2016 to 2016]	9	0 [−1, 16]	1	9.9 [9.9, 9.9]
East Africa	41	2016 [2016 to 2017]	33	2 [−1, 7]	3	17.5 [8.7, 19.7]
North America	33	2014 [2010 to 2015]	22	11 [−35, 40]	0	‐
Southern Africa	34	2016 [2016 to 2016]	34	2 [1, 2]	0	‐
West Africa	5	2017 [2017 to 2017]	0	‐	5	6.7 [6.4, 8]
Health facility type						
Primary (health center)	92	2016 [2016 to 2016]	79	2 [0, 2]	3	6.4 [5.7, 19.7]
District hospital	17	2016 [2016 to 2016]	14	2 [−1, 3]	0	‐
Regional/provincial or teaching hospital	69	2015 [2014 to 2016]	45	1 [−9, 12]	11	8 [3.9, 12.7]
Sector						
Public	148	2016 [2015 to 2016]	113	2 [0, 5]	13	8 [5.6, 12.7]
Private	30	2015 [2011 to 2016]	25	0 [−50, 2]	1	6.7 [6.7, 6.7]
Facility location						
Urban/Mostly urban	128	2016 [2014.5 to 2016]	91	0 [−6, 5]	13	6.7 [5.6, 9.9]
Rural/Mostly rural	50	2016 [2016 to 2016]	47	2 [2, 3]	1	19.7 [19.7, 19.7]
Country income group						
Low income	54	2016 [2016 to 2017]	49	2 [1, 2]	5	12.7 [9.9, 17.5]
Lower‐middle income	42	2016 [2016 to 2016]	31	1 [−1, 3]	5	6.4 [5.7, 6.7]
Upper‐middle income	27	2016 [2015 to 2016]	24	0 [−3.5, 7]	1	3.9 [3.9, 3.9]
High income	55	2015 [2012 to 2015]	34	−1 [−21, 27]	3	5.6 [0.6, 16.1]
PEPFAR‐supported country						
No	77	2015 [2014 to 2016]	50	−1 [−13, 21]	6	4.7 [3.3, 12.7]
Yes	101	2016 [2016 to 2016]	88	2 [0, 2]	8	8.4 [6.6, 13.7]
GFATM‐supported country						
No	66	2015 [2013 to 2015]	44	−1 [−12.5, 22]	3	5.6 [0.6, 16.1]
Yes	112	2016 [2016 to 2016]	94	2 [0, 2]	11	8.0 [5.7, 12.7]
Year of national Treat All adoption						
2012 (2 countries)	33	2014 [2010 to 2015]	22	11 [−35, 40]	‐	‐
2013 (2 countries)	8	2013 [2013 to 2015]	8	−0.5 [−9, 18.5]	‐	‐
2014 (2 countries)	6	2015 [2014 to 2016]	5	12 [0, 12]	‐	‐
2015 (2 countries)	16	2015 [2014.5 to 2015.5]	10	−1 [−7, 5]	‐	‐
2016 (16 countries)	95	2016 [2016 to 2016]	89	2 [0, 2]	‐	‐
2017 (2 countries)	4	2013.5 [2011.5 to 2015.5]	4	−40.5 [−63, −18]	‐	‐
Timing of national Treat All adoption^b^						
Before WHO recommendation	63	2015 [2013 to 2015]	45	0 [−9, 22]	‐	‐
After WHO recommendation	99	2016 [2016 to 2016]	93	2 [0, 2]	‐	‐
Treat All not adopted nationally	16	2016.5 [2016 to 2017]	‐	‐	14	7.4 [5.6, 12.7]

^a^Sites with known month and year of Treat All introduction; ^b^timing relative to WHO recommendation of September 2015.

**Figure 2:**
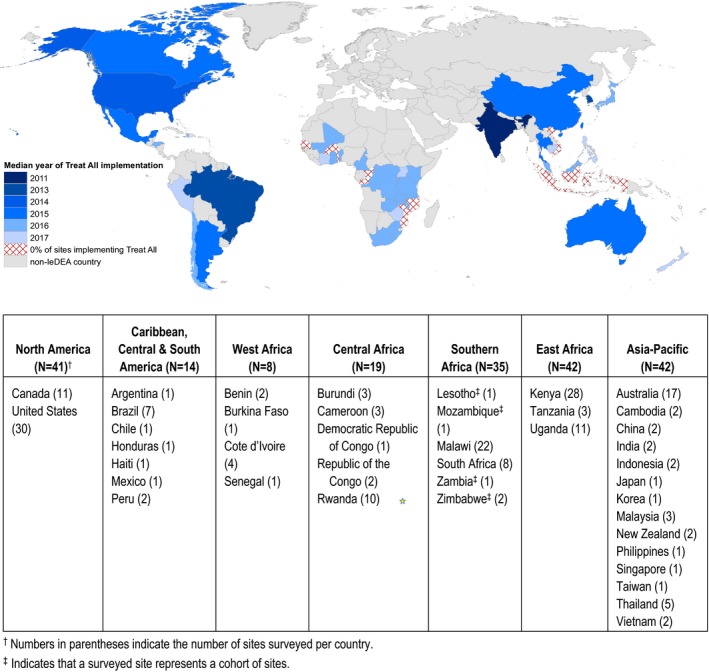
Median year of site‐level implementation of Treat All among 201 IeDEA sites.

At the country level, the median year of site‐level Treat All introduction ranged from 2011 to 2017 (Figure 2 and Table S1). In several countries where Treat All had not been adopted nationally at the time of the survey (e.g. Burkina Faso, Republic of the Congo, Senegal, Mozambique, Indonesia and Vietnam), none of the surveyed sites had introduced Treat All.

Almost all sites (178/187, 95%) reporting implementation of Treat All specified the year of site‐level introduction, and 81% (152/187) reported both month and year of site‐level introduction. Just over one‐third (35%) of sites reported that they began initiating all patients on ART regardless of immune status or clinical disease stage prior to the adoption of Treat All in national guidelines (Table 1). Site‐level introduction of Treat All prior to national guideline adoption was significantly more common among district, regional/provincial and teaching hospitals (37/81, or 46%) than primary‐level health centres (22/89, or 25%; *p* = 0.006) and at sites in urban areas (54/122, or 44%), compared with rural sites (5/48 or 10%; *p* < 0.0001). All Treat All‐implementing sites in the West Africa region (100%) and almost two‐thirds of sites in the Asia‐Pacific region reported that site‐level introduction of Treat All preceded national guideline adoption, as did half of the surveyed sites in the Caribbean, Central and South America and about one‐third of sites in the East Africa and North America regions. In contrast, no sites in Southern Africa and few sites in Central Africa reported implementing Treat All prior to national guideline adoption.

### 3.3 Time from national adoption of Treat All to site‐level introduction

In countries where Treat All had been adopted nationally, 138/175 (79%) sites reported both month and year of Treat All introduction. Among these sites, the median time lag between national guideline adoption and site‐level introduction was 1 month (IQR: −1 to 4 months) (Table 2). Intervals between national guideline adoption and site‐level introduction were significantly longer at rural sites (median time 2 months; IQR: 2 to 3 months) compared with urban sites (median time zero months; IQR: −6 to 5 months; *p* < 0.001) and at public sector sites (median time 2 months; IQR: 0 to 5 months) compared with private sector facilities (median time 0 months; IQR: −50 to 2 months; *p* = 0.0046). The time to site‐level introduction of Treat All was also longer in low‐ and lower‐middle‐income countries (median time 2 months; IQR: 0 to 2.5 months), compared with high‐ and upper‐middle‐income countries (median time 0 months; IQR: −9 to 16 months) and in PEPFAR‐supported countries (median time 2 months; IQR: 0 to 2 months) compared with non‐PEPFAR‐supported countries (median time −1 month; IQR: −13 to 21 months); however, these differences were not statistically significant, given wide variation in the timing of Treat All in high‐ and upper‐middle‐income countries. Similarly, the time to site‐level introduction of Treat All was longer at health centres (median time 2 months; IQR: 0 to 2 months), compared with district, regional/provincial and teaching hospitals (median time one month; IQR: −4 to 12 months) (differences not statistically significant).

In countries where national Treat All policies had not yet been adopted, 14/26 sites that were treating all patients irrespective of CD4+ counts or other symptoms reported that they had been initiating all patients on ART for a median period of seven months at the time of the survey (IQR: −13 to −6 months).

### 3.4 ART initiation practices and viral load monitoring at Treat All‐implementing sites

Approximately two‐thirds (66%) of sites implementing Treat All reported that they typically conduct one to two adherence counselling sessions prior to initiating patients on ART, while 24% (45/187) reported providing three or more such sessions (Table 3), and 10% (19/187) reported that patients typically have no pre‐ART counselling sessions. Private sector sites were significantly more likely than public sector sites to report no adherence counselling sessions prior to ART initiation (29% vs. 6%; *p* < 0.001), as were sites in urban areas compared to rural areas (13% vs. 2%; *p* = 0.03). Sites in high‐income countries and countries not supported by PEPFAR or the Global Fund were also significantly more likely to report that patients typically have no pre‐ART counselling sessions (21% to 28% vs. 1% to 2%; *p* < 0.05). Among the 14 sites that had not yet introduced Treat All, half (7/14) reported that they typically conduct one to two counselling sessions before initiating patients on ART, with six sites reporting that patients typically have three or more counselling sessions prior to initiating treatment.

**Table 3:** ART initiation practices and viral load testing capacity at 187 IeDEA sites implementing Treat All

	Counseling sessions prior to ART initiation			Timing of ART initiation			Viral load^a^ testing routinely available N (%)
	0 sessions N (%)	1 to 2 sessions N (%)	≥3 sessions N (%)	Same day start N (%)	1 to 14 days N (%)	2 to 4 weeks or >1 month N (%)	
All sites	19 (10.2%)	123 (65.8%)	45 (24.1%)	73 (39%)	71 (38%)	43 (23.0%)	129 (69%)
IeDEA region							[*p* < 0.0001]
Asia‐Pacific	3 (8.3%)	27 (75%)	6 (16.7%)	3 (8.3%)	19 (52.8%)	14 (38.9%)	33 (91.7%)
Caribbean, Central and South America	0 (0%)	14 (100%)	0 (0%)	5 (35.7%)	4 (28.6%)	5 (35.7%)	12 (85.7%)
Central Africa	0 (0%)	8 (47.1%)	9 (52.9%)	4 (23.5%)	13 (76.5%)	0 (0%)	5 (29.4%)
East Africa	0 (0%)	19 (45.2%)	23 (54.8%)	26 (61.9%)	16 (38.1%)	0 (0%)	23 (54.8%)
North America	15 (38.5%)	22 (56.4%)	2 (5.1%)	11 (28.2%)	10 (25.6%)	18 (46.2%)	38 (97.4%)
Southern Africa	1 (2.9%)	28 (82.4%)	5 (14.7%)	21 (61.8%)	8 (23.5%)	5 (14.7%)	15 (44.1%)
West Africa	0 (0%)	5 (100%)	0 (0%)	3 (60%)	1 (20%)	1 (20%)	3 (60.0%)
Health facility type			[*p* = 0.006]			[*p* = 0.036]	[*p* < 0.0001]
Primary (health center)	12 (12.4%)	61 (62.9%)	24 (24.7%)	39 (40.2%)	39 (40.2%)	19 (19.6%)	55 (56.7%)
District hospital	0 (0%)	7 (41.2%)	10 (58.8%)	12 (70.6%)	3 (17.6%)	2 (11.8%)	11 (64.7%)
Regional/provincial or teaching hospital	7 (9.6%)	55 (75.3%)	11 (15.1%)	22 (30.1%)	29 (39.7%)	22 (30.1%)	63 (86.3%)
Sector			[*p* = 0.001]			[*p* = 0.019]	[*p* = 0.142]
Public	10 (6.4%)	104 (66.7%)	42 (26.9%)	66 (42.3%)	60 (38.5%)	30 (19.2%)	104 (66.7%)
Private	9 (29%)	19 (61.3%)	3 (9.7%)	7 (22.6%)	11 (35.5%)	13 (41.9%)	25 (80.6%)
Facility location			[*p* = 0.033]			[*p* < 0.0001]	[*p* < 0.0001]
Urban/mostly urban	18 (13.2%)	89 (65.4%)	29 (21.3%)	40 (29.4%)	54 (39.7%)	42 (30.9%)	110 (80.9%)
Rural/mostly rural	1 (2%)	34 (66.7%)	16 (31.4%)	33 (64.7%)	17 (33.3%)	1 (2%)	19 (37.3%)
Country income group			[*p* < 0.0001]			[*p* < 0.0001]	[*p* < 0.0001]
Low income	1 (1.9%)	39 (72.2%)	14 (25.9%)	23 (42.6%)	28 (51.9%)	3 (5.6%)	21 (38.9%)
Lower‐middle income	0 (0%)	20 (45.5%)	24 (54.5%)	31 (70.5%)	13 (29.5%)	0 (0%)	23 (52.3%)
Upper‐middle income	1 (3.6%)	24 (85.7%)	3 (10.7%)	5 (17.9%)	13 (46.4%)	10 (35.7%)	26 (92.9%)
High income	17 (27.9%)	40 (65.6%)	4 (6.6%)	14 (23%)	17 (27.9%)	30 (49.2%)	59 (96.7%)
PEPFAR‐supported country			[*p* < 0.0001]			[*p* < 0.0001]	[*p* < 0.0001]
No	18 (21.4%)	61 (72.6%)	5 (6%)	18 (21.4%)	28 (33.3%)	38 (45.2%)	80 (95.2%)
Yes	1 (1%)	62 (60.2%)	40 (38.8%)	55 (53.4%)	43 (41.8%)	5 (4.9%)	49 (47.6%)
GFATM‐supported country			[*p* < 0.0001]			[*p* < 0.0001]	[*p* < 0.0001]
No	17 (23.6%)	51 (70.8%)	4 (5.6%)	17 (23.6%)	21 (29.2%)	34 (47.2%)	70 (97.2%)
Yes	2 (1.7%)	72 (62.6%)	41 (35.7%)	56 (48.7%)	50 (43.5%)	9 (7.8%)	59 (51.3%)
Year of national Treat All adoption							[*p* < 0.0001]
2012 (2 countries)	15 (38.5%)	22 (56.4%)	2 (5.1%)	11 (28.2%)	10 (25.6%)	18 (46.2%)	38 (97.4%)
2013 (2 countries)	0 (0%)	8 (100%)	0 (0%)	3 (37.5%)	2 (25.0%)	3 (37.5%)	8 (100%)
2014 (2 countries)	1 (16.7%)	4 (66.7%)	1 (16.7%)	0 (0%)	4 (66.7%)	2 (33.3%)	6 (100%)
2015 (2 countries)	2 (12.5%)	14 (87.5%)	0 (0%)	1 (6.3%)	6 (37.5%)	9 (56.3%)	15 (93.8%)
2016 (16 countries)	1 (1.0%)	57 (59.4%)	38 (39.6%)	52 (54.2%)	38 (39.6%)	6 (6.3%)	45 (46.9%)
2017 (2 countries)	0 (0%)	3 (75.0%)	1 (25.0%)	1 (25.0%)	2 (50.0%)	1 (25.0%)	4 (100%)
Treat All not adopted nationally^b^ (15 countries)	0 (0%)	15 (83.3%)	3 (16.7%)	5 (27.8%)	9 (50.0%)	4 (22.2%)	13 (72.2%)
Timing of national Treat All adoption^c^			[*p* < 0.0001]			[*p* < 0.0001]	[*p* < 0.0001]
Before WHO recommendation	18 (26.1%)	48 (69.6%)	3 (4.3%)	15 (21.7%)	22 (31.9%)	32 (46.4%)	67 (97.1%)
After WHO recommendation	1 (1%)	62 (60.2%)	40 (38.8%)	53 (53.0%)	40 (40.0%)	7 (7.0%)	49 (49.0%)
Treat All not adopted nationally	0 (0%)	13 (86.7%)	2 (13.3%)	5 (27.8%)	9 (50.0%)	4 (22.2%)	13 (72.2%)

^a^Quantitative PCR or viral load assay available for routine use; ^b^sites in countries that adopted Treat All in 2017 after the survey was completed counted among sites where Treat All was not yet adopted nationally; ^c^timing relative to WHO recommendation of September 2015.

Among 187 sites implementing Treat All, 77% reported initiating patients on ART within 14 days of establishing treatment eligibility (Table 3). Same‐day ART initiation was more commonly reported by sites in the East, Southern, and West Africa regions (60% to 62%), as well as by district hospitals (12/17, or 71%) and public sector sites (66/156, or 42%). In contrast, almost half of private sector facilities (13/31, or 42%) reported that patients did not initiate ART until two to four weeks or longer after confirming HIV diagnosis, as did sites in high‐income countries (30/61, or 49%), in countries not supported by PEPFAR (38/84, or 45%) or the Global Fund (34/72, or 47%), and in countries where Treat All was adopted in national guidelines before WHO's 2015 recommendation (23/69, or 46%). More than one quarter (11/43 or 26%) of the sites reporting that they initiate patients on ART two to four weeks or longer after confirming diagnosis also reported that typically they do not conduct adherence counselling sessions prior to ART initiation; in contrast, only 7% (5/72) sites reporting same day ART initiation reported that their patients typically do not attend any pre‐ART counselling sessions.

More than two‐thirds of sites implementing Treat All (129/187) reported that viral load testing was available as part of routine care of patients at the site. Viral load testing capacity was nearly universal among sites in high‐income (59/61, or 97%) and upper‐middle‐income countries (26/28, or 93%). However, only 39% (21/54) of sites in low‐income countries and 52% (23/44) of sites in lower‐middle‐income countries reported that viral load testing was available as part of routine patient care. Capacity for routine viral load monitoring was also significantly more common among urban sites compared with rural sites (110/136 (81%) vs. 19/51 (37%); *p* < 0.0001); and in hospitals (district, regional/provincial or teaching hospitals) compared with health centres (74/90 (82%) vs. 55/97 (57%); *p* < 0.001).

## 4 Discussion

Surveying 201 adult HIV treatment sites that participate in the global IeDEA collaboration across 41 countries, this study found that the vast majority of sites had begun initiating all patients on ART by mid‐2017, regardless of immune status or clinical disease stage. Site‐level implementation of Treat All is almost universal in countries that have incorporated WHO's 2015 recommendation into national guidelines. Previous research has highlighted various logistical barriers and health system constraints, such as guideline dissemination, training of health personnel, drug stockouts and gaps in laboratory capabilities, which contributed to delays in implementing prior, CD4 count–based treatment guidelines 10, 12, 23. However, our study found that the time from national adoption to site‐level introduction was relatively rapid across IeDEA‐participating sites in 41 countries, with a median time‐to‐implementation of one month. In countries where Treat All was adopted in national guidelines in 2017, all IeDEA sites had begun implementing WHO's guidance in advance of national guideline changes. Likely reasons for the rapid roll‐out of Treat All at the service delivery level may include temporal improvements in the capacity to deliver ART across health systems, as well as the possibility that Treat All simplifies the provision of HIV treatment in low‐resourced health systems (38, 39).

In countries that officially adopted the Treat All policy prior to WHO's 2015 guidance, there was greater between‐site variation in the timing of site‐level introduction of Treat All, with implementation lags ranging from one to five years after national policy adoption. In these predominately high‐income countries, variation in site‐level introduction may reflect heterogeneous health policy environments, decentralized health systems, and a higher proportion of private‐sector sites operating autonomously. Earlier site‐level adoption of Treat All in these countries may reflect the feasibility of expanding HIV treatment, given lower HIV prevalence, higher‐resourced health systems, and stronger logistics systems in these settings 23, 24, whereas later site‐level introduction in these countries may reflect barriers, such as gaps in providers’ knowledge, or lingering provider concerns about potential negative consequences, such as the emergence of resistance, treatment side effects, increased sexual risk‐taking and lack of patient‐readiness 25, 26, 27, 28. In contrast, there was less between‐site variation in time‐to‐implementation in countries adopting Treat All after WHO's 2015 recommendation, especially in low‐/lower‐middle‐income countries supported by PEPFAR and/or the Global Fund, suggesting that site‐level roll‐out of Treat All may be more uniform in countries receiving support from these and other donors 29.

The majority of surveyed sites in low‐ and lower‐middle‐income countries reported typically starting patients on ART within 14 days of eligibility ascertainment, which is promising, given evidence that rapid ART initiation leads to improved clinical outcomes 30, 31. Surprisingly, in high‐income countries and countries with earlier national adoption of Treat All, sites were more likely to report that patients generally initiate ART two to four weeks or more after diagnosis is confirmed, possibly reflecting heterogeneous provider practices in these contexts, genotypic resistance testing, organ function testing, or delays incurred in the coordination of multiple stakeholders, including service delivery and insurance providers, prior to treatment initiation 32, 33, 34, 35. Interestingly, sites that reported longer times between enrolment and ART initiation were also more likely to report that their patients typically had no treatment readiness counselling sessions prior to treatment initiation, suggesting these are not a likely source of delay in initiating patients on ART.

Our study provides important early data assessing the timing of Treat All introduction at the service delivery level, as well as the interval between national guideline adoption and site‐level introduction. With a sample of 201 sites serving adult HIV patients across 41 countries, the IeDEA site assessment reflects the status of Treat All rollout among a large and diverse sample of HIV care and treatment sites across different regions, countries and types of facilities. While almost all sites reported universal HIV treatment as the standard of care, we found considerable variation in site‐level practices related to rapid ART initiation, with private sector sites and sites in high‐resource settings more likely to report longer times to treatment initiation. Consistent with other research 16, 36, 37, 38, we also found considerable variation in capacity for viral load monitoring, and almost one‐third of the sites implementing Treat All indicated that they could not routinely request or perform viral load testing. Such gaps were particularly prevalent among sites located in rural areas and in low‐income countries, as well as in countries where Treat All was adopted more recently. Our findings also underscore the need to identify and address bottlenecks, including health care payment systems, administrative processes, and provider practices, that contribute to delays in initiating patients on treatment, as well as those contributing to suboptimal levels of treatment adherence and sustained viral suppression (e.g. stockouts of antiretroviral medications and capacity gaps related to viral load and resistance monitoring). Programme monitoring and research is also needed on implementation barriers at sites that continue to operate under previous guidelines.

Several study limitations should be noted. The survey data were self‐reported. Accordingly, in some instances, recall bias and social desirability bias may have led to inaccurate responses and/or responses more closely aligned with national treatment guidelines and norms than true practice. HIV care and treatment sites participating in IeDEA are primarily public‐sector health facilities, and they comprise a heterogeneous mix of academic and community‐based hospitals and health centres 16. However, IeDEA‐participating sites are unlikely to be representative of all HIV care clinics in a given country. Moreover, in countries where one site completed the survey on behalf of a larger network of sites, there may be greater heterogeneity in site‐level practices than is reflected in the survey results. Accordingly, general practice related to Treat All implementation in each country may differ from the results reported here, and the timing of site‐level Treat All introduction and estimates of the median time from national guideline adoption and site‐level introduction may mask in‐country variation.

## 5 Conclusions

Data from this survey indicate that by mid‐ to late‐2017, the Treat All strategy was being implemented across most IeDEA sites in 41 countries, including rural, primary‐level health facilities in low‐resource settings. Further research is needed to assess patient outcomes and the quality and effectiveness of HIV‐related care under Treat All and to identify bottlenecks that contribute to ongoing delays in ART initiation and other suboptimal patient outcomes. However, the accelerating roll‐out of Treat All at HIV care and treatment sites across IeDEA is promising for the achievement of UNAIDS’ 95‐95‐95 targets to end the AIDS epidemic.

## Competing interests

KNA serves on the scientific advisory board for Trio Health. All authors declare that they have no competing interests.

## Authors’ contributions

EB, SND, FM, DN and CWW developed the survey questionnaire. SND and FM designed the survey in REDCap, and FM coordinated overall data collection. DN conceptualized the project, and EB performed the data analysis and wrote the initial draft of the manuscript. FM, SND, OT, CWW, GS, JR, AF, MC, AP, BSM, FZ, KNA, CM, ADK, MY and DN contributed to the interpretation of findings and manuscript revisions, and all authors read and approved the final manuscript.

## Supporting information


**Table S1.** Date of national adoption of Treat All, median year, and time‐to‐implementation at IeDEA sites operating under Treat All at the time of the survey.

## Appendix 1 IeDEA Asia Pacific

### 1.1

Azar Karminia, Annette H. Sohn. Australia: Debbie Allen, Mark Bloch, Susan Boyd, Katherine Brown, Jess Costa, William Donohue, Manoji Gunathilake, Jennifer Hoy, Karen MacRae, Richard Moore, Norman Roth, Diane Rowling, Julie Silvers, David J. Smith, David Sowden, David Templeton, Rick Varma, Ian Woolley, David Youds. Cambodia: Somanithd Chhay Meng, Bun Vannary. China: Yun Ting Chan, Wilson Lam, Man Po Lee, Han Ning, Yu Po Chu Pansy, Fujie Zhang. India: N. Kumarasamy, Sanjay Pujari. Indonesia: Nia Kurniati, Tuti Parwati Merati, Dina Muktiarti, Wayan Sandhi Parwata, Made Ratni, Ni Made Dewi Dian Sukmawati, Dian Sulistya Putu Diah Vedaswari, Ketut Dewi Kumara Wati, Evy Yunihastuty. Japan: Junko Tanuma. New Zealand: Graham Mills, Nigel Raymond. Philippines: Rossana Ditangco. Singapore: Ohnmar Seinn Papa, Ng Oon Tek. Malaysia: Dr. Ah‐neez, Raja Azwa, Prof. Dato, Fauziah Daud, Wong Ke Juin, Adeeba Binti Kamarulzaman, Nik Khairulddin, Chong Meng Li, Fong Siew Moy, Raja Iskandar Shah, Wong Peng Shyan, Benedict Sim, Jamal Mohamed Thahira, Koh Mia Tuang, Nik Yusoff. South Korea: Jun Yong Choi. Taiwan: Yu‐Jiun Chan, Chih‐Sheng Huang, Wong Wing‐Wai. Thailand: Anchalee Avihingsanon, Kulkanya Chokephaibulkit, Rawiwan Hansudewechakul, Benjhawan Khumcha, Suwimon Khusuwan, Sasisopin Kiertiburanakul, Pagakrong Lumbiganon, Alan Maleesatharn, Jutarat Praparattanapan, Thanyawee Puthanakit, Sirintip Sricharoenchai, Tavitiya Sudjaritruk, Suporn Watanaporn. Vietnam: Dr. Vu Thien An, Do Duy Cuong, Bùi Thu Hằng, Bùi Vũ Huy, Du Tuan Quy, Lam Nguyen Van.

### Central Africa IeDEA

Burundi: Agathomfue Baragunzwa, Dévote Gakima, Gloria Ingabire, Floride Kankinoi, Risase Scholastique Manyundo, Celestin Misago, Thierry Nahimana, Pélagie Nimbona, Felicite Ntirampeba, Christella Twizere. Cameroon: Rogers Ajeh, Amadou Djenabou, Anastase Dzudie, Alice Ndelle Ewanoge, Edmond Tchassem. Democratic Republic of Congo: Therese Bampapa, Patricia Lelo, Faustin Kitetele, Marie Paul, Amida Tytyna. Republic of the Congo: Maryse Akolbout, Parfait Bitsindou, Merlin Diafouka, Adolphe Mafoua, Nadine Mahinga, Ella Moudila, Antoinette Moutoula, Ulrich Ndala, Dominique Mahambou Nsonde. Rwanda: Josephine Ayinkamiye, Chantal Dusabe, Theogene Hakizimana, Gilbert Mbaraga, Joyce Mukamana, Sandrine Mukantwali, Athanase Munyaneza, Anthere Murangwa, J. Claude Musenguwera, Marie Immanculee Ngutegure, Fidele Ntarambirwa, Diane Nyiransabimana, Jean d'Amour Sinayobye, Yvonne Tuyishimire, Olive Uwamahoro, Olive Uwamahoro, Habumuremyi Viateur, Sugira Vincent.

### IeDEA East Africa

Yee Yee Kuhn, Beverly Musick, Israel Rodriguez, Kara Wools‐Kaloustian, Constantin Yiannoutsos. Kenya: Esinasi Akajoroit, Peter Ariya, Meshack Atsimale, Zeruya Barua, Oscar Busaka, Elizabeth Bukusi, Valentine Chebor, Timothy Chemweno, John Chirchir, Lameck Diero Sagida Esendi, Aisha Fwamba, Anne Mmella, Eunice Githumbi, Marcia Nasimiyu Hussein, Xavier Kandie, Martha Kemunto, Elizabeth Khaemba, Mary Kipchumba, Emily Koech, Caroline Kosgei, Barasa Laundrick, Ruth Merongo, Patricia Mochotto, Consolata Munyisi, Lilian Ndakalu, William Okoth Ochieng, Paul Odalo, Wicklife Okumu, Lilian Omari, Alphoce Omondi, Lydia Osia, Magret Owino, Maureen Oyoo, Doris Pepela, Millicent Rono, Omar Simon, Angie Tenge, Mary Too, Modesta Toto, Cathrine Towett, Kennedy Wawire. Tanzania: Mensaria Kimambo, Ester Kinyota, Rita Lyamuya, Julia Mathias, Athuman Ramadhan Mfuko, Denna Michael, Kapella Zacharia Ngonyani, Charles Nyaga, G.R. Somi, Mark S. Urassa. Uganda: James Batte, Mwebesa Bosco Bwana, Barbara Castelnuovo, Michael Kanyesigye, Alice Kisakye, Fred Nalugoda, Haruna Semuwemba, John Ssali, Matthew Ssemakadde.

### IeDEA Caribbean, Central and South America (CCASAnet)

Jessica Castilho. Argentina: Carina Cesar. Brazil: Paulo Ricardo de Alencastro, Eduardo Luiz Barbosa, Carlos Brites, Renata Caricol, Fabiana Bononi do Carmo, Lara Esteves Coelho, Maria Mercedes Escuder, Denize Lotufo Estevam, Flavia Gomes Faleiro Ferreira, Alexandre Gonçalves, Aída de Fátima Barbosa Gouvêa, Maria Leticia Rodrigues Ikeda, Artur O. Kalichman, Daisy Maria Machado, Simone Queiroz, Rosa de Alencar Souza, Regina Célia Succi, Kátia Valeska Trindade, Unai Tupinambás. Chile: Marcelo Wolff. Haiti: Vanessa Rouzier. Honduras: Denis Padgett. Mexico: Brenda Crabtree. Peru: Carlos Eduardo Verne Martin, Fernando Mejia.

### IeDEA North America (NA‐ACCORD)

Canada: Benny Chang, Brenda Done, Larry Gabe, John Gill, Kevin Gough, Gail Howlett, Marin Klein, Judy Latendre‐Paquette, Victor Leung, Paul MacPhee, P. MacPherson, Raj Maharaj, Lorna Carrasco Medina, Suzanne Page, Costas Pexos, Anita Rachlis, Kate Salters, Sherine Sterling. USA: Stephen Boswell, Greer Burkholder, Gisela Cesteros. Kalliope Chagaris, Rosa Franklin, Aimee Freeman, Jack Fuhrer, Cynthia L. Gilbert, Matthew Goetz, Chris Grasso, Michael Horberg, Robert F Hunter‐Mellado, Rita Kell, Mari Kitahata, Daniel Klein, Ken Levine, Vincent Marconi, Christopher Mathews, Angel M. Mayor, Catherine McGowan, Richard Moore, Sonia Napravnik, Richard Novak, Kris Ann Oursler, Shellier Ramos, Benigno Rodriguez, Maria C. Rodriguez, Michael Silverberg, Michael S. Simberkoff, Mohit Varshney, Douglas Ward, Barb Widick, Bienvenido G. Yangco.

### IeDEA Southern Africa

Morna Cornell, Mary‐Ann Davies, Lilian Smith, Per Maximilian von Groote. Lesotho: Josephine Muhairwe. Malawi: Steve Balakasi, Quietus Banda, Getrude Kalepa, Bauman, Andrew Bello, J.W. Bulla, Maria Chigeda, Joyce Chikaphupha, Flora Chikwekwere, Jack Kachoka, Allan Kapito, Alinafe Nathan Katondo, Molly Kumwenda, Felix Phewa Labein, Ronald Magombo, Bridget Malumbe, I. Makuwira, Patricia Marico, Betha Masangale, Angella Mchiela, Dan Midian, Kezia Phiri, Mary Tambe, Baid Thomas, Charles Thomson. Mozambique: Jonas Hector. South Africa: Anna Cross, Siphephelo Dlamini, Brian Eley, Jonathan Euvrard, Geoffrey Fatti, Katherine Hilderbrand, Marvin Hsiao, Michael Mpye, Hans Prozesky, Gary Reubenson, Lesley Rose, Shobna Sawry, Nosisa Sibambo, Karl Technau. Zambia: Michael Vinikoor. Zimbabwe: Cleophas Chimbetete, Kamelia Kamenova.

### IeDEA West Africa

Eric Balestre, Valeriane Leroy, Karen Malasteste. Benin: Marcel Zannou Djimon, Marcelline D'Almeida, Ghislaine Hounhoui, Michee Assogba. Burkina Faso: Armel Poda, Jacques Zoungrana, Issouf Yaméogo, Achille Tapsoba. Cote d'Ivoire: Sidibé Abdelh, Clarisse Amani Bosse, Mamoudou Diabaté, Tanoh Kassi François Eboua, Madeleine Amorissani Folquet, Denise Hawelander, Mamadou Konaté, Kouadio Kouakou, Dohoun Lambert, Albert Kla Minga, Marie Sylvie N'gbeche, Aristophane Tanon, Abo Yao. Ghana: Lorna Renner. Mali: Dr. Clémentine N'Diaye, Mme Alima Berthé. Senegal: Moussa Seydi, Judicaël Tine. Togo: Takassi Ounoo Elom, Benjamin Kariylare, Akessiwe Patassi.

## References

[1] World Health Organization . Guideline on when to start antiretroviral therapy and on pre‐exposure prophylaxis for HIV. Geneva: World Health Organization; 2015. 26598776

[2] INSIGHT START Study Group , Lundgren JD , Babiker AG , Gordin F , Emery S , Grund B , Sharma S , et al. Initiation of antiretroviral therapy in early asymptomatic HIV infection. N Engl J Med. 2015;373(9):795–807. 26192873 10.1056/NEJMoa1506816 PMC4569751

[3] TEMPRANO ANRS Study Group , Danel C , Moh R , Gabillard D , Badje A , Le Carrou J , et al. A Trial of early antiretrovirals and isoniazid preventive therapy in Africa. N Engl J Med. 2015;373(9):808–22. 26193126 10.1056/NEJMoa1507198

[4] Granich RM , Gilks CF , Dye C , De Cock KM , Williams BG . Universal voluntary HIV testing with immediate antiretroviral therapy as a strategy for elimination of HIV transmission: a mathematical model. Lancet. 2009;373(9657):48–57. 19038438 10.1016/S0140-6736(08)61697-9

[5] Cohen MS , Chen YQ , McCauley M , Gamble T , Hosseinipour MC , Kumarasamy N , et al. Prevention of HIV‐1 infection with early antiretroviral therapy. N Engl J Med. 2011;365(6):493–505. 21767103 10.1056/NEJMoa1105243 PMC3200068

[6] Baeten JM , Donnell D , Ndase P , Mugo NR , Campbell JD , Wangisi J , et al. Antiretroviral prophylaxis for HIV prevention in heterosexual men and women. N Engl J Med. 2012;367(5):399–410. 22784037 10.1056/NEJMoa1108524 PMC3770474

[7] UNAIDS . 90‐90‐90 An ambitious treatment target to help end the AIDS epidemic. Geneva, Switzerland: UNAIDS; 2014.

[8] Joint United Nations Programme on HIV/AIDS (UNAIDS) . Fast‐Track: ending the AIDS epidemic by 2030. Geneva. 2014.

[9] Gupta S , Granich R . When will sub‐Saharan Africa adopt HIV treatment for all? Southern Afr J HIV Med. 2016;17(1):1–6. 10.4102/sajhivmed.v17i1.459 PMC5843218 29568615

[10] Ambia J , Renju J , Wringe A , Todd J , Geubbels E , Nakiyingi‐Miiro J , et al. From policy to practice: exploring the implementation of antiretroviral therapy access and retention policies between 2013 and 2016 in six sub‐Saharan African countries. BMC Health Serv Res. 2017;17(1):758. 29162065 10.1186/s12913-017-2678-1 PMC5698969

[11] Tlhajoane M , Masoka T , Mpandaguta E , Rhead R , Church K , Wringe A , et al. A longitudinal review of national HIV policy and progress made in health facility implementation in Eastern Zimbabwe. Health Res Policy Syst. 2018;16(1):92. 30241489 10.1186/s12961-018-0358-1 PMC6150955

[12] Mwangome MN , Geubbels E , Wringe A , Todd J , Klatser P , Dieleman M . A qualitative study of the determinants of HIV guidelines implementation in two south‐eastern districts of Tanzania. Health Policy Plan. 2017;32(6):825–34. 28369374 10.1093/heapol/czx023 PMC5448494

[13] World Health Organization (WHO) . Treat all: Policy adoption and implementation status in countries: Fact sheet. Geneva, Switzerland: World Health Organization, Department of HIV/AIDS; 2017.

[14] Ford N , Ball A , Baggaley R , Vitoria M , Low‐Beer D , Penazzato M , et al. The WHO public health approach to HIV treatment and care: looking back and looking ahead. Lancet Infect Dis. 2018;18:e76–86. 29066132 10.1016/S1473-3099(17)30482-6

[15] IeDEA . International Epidemiologic Databases to Evaluate AIDS Bern, Switzerland: the Institute of Social and Preventive Medicine (ISPM), University of Bern. [cited 2018 June 1]. Available from: https://www.iedea.org/

[16] Fritz CQ , Blevins M , Lindegren ML , Wools‐Kaloutsian K , Musick BS , Cornell M , et al. Comprehensiveness of HIV care provided at global HIV treatment sites in the IeDEA consortium: 2009 and 2014. J Int AIDS Soc. 2017;20(1):20933. 28364561 10.7448/IAS.20.1.20933 PMC5463912

[17] Duda SN , Farr AM , Lindegren ML , Blevins M , Wester CW , Wools‐Kaloustian K , et al. Characteristics and comprehensiveness of adult HIV care and treatment programmes in Asia‐Pacific, sub‐Saharan Africa and the Americas: results of a site assessment conducted by the International epidemiologic Databases to Evaluate AIDS (IeDEA) Collaboration. J Int AIDS Soc. 2014;17(1):19045. 25516092 10.7448/IAS.17.1.19045 PMC4268491

[18] Harris PA , Taylor R , Thielke R , Payne J , Gonzalez N , Conde JG . Research electronic data capture (REDCap) – a metadata‐driven methodology and workflow process for providing translational research informatics support. J Biomed Inform. 2009;42(2):377–81. 18929686 10.1016/j.jbi.2008.08.010 PMC2700030

[19] International Association of Providers of AIDS Care (IAPAC) . Global HIV Policy Watch. Washington DC: IAPAC; 2017[cited 2018 Aug 10]. Available from: http://www.hivpolicywatch.org/index.html

[20] The World Bank . World Bank list of economies. 2017[cited 2017 Nov 10]. Available from: https://datahelpdesk.worldbank.org/knowledgebase/articles/906519-world-bank-country-and-lending-groups

[21] U.S. President's Emergency Plan for AIDS Relief (PEPFAR) . PEPFAR Bilateral Countries: Office of U.S. Global AIDS Coordinator and the Bureau of Public Affairs, U.S. State Department. [cited 2018 Aug 1]. Available from: https://www.pepfar.gov/countries/bilateral/index.htm

[22] The Global Fund to Fight AIDS Tuberculosis and Malaria . The Global Fund Data Service: The Global Fund. [cited 2019 Mar 20]. Available from: https://data-service.theglobalfund.org/downloads

[23] Bigna JJ , Plottel CS , Koulla‐Shiro S . Challenges in initiating antiretroviral therapy for all HIV‐infected people regardless of CD4 cell count. Infect Dis Poverty. 2016;5(1):85. 27593965 10.1186/s40249-016-0179-9 PMC5011352

[24] Gupta S , Granich R , Suthar AB , Smyth C , Baggaley R , Sculier D , et al. Global policy review of antiretroviral therapy eligibility criteria for treatment and prevention of HIV and tuberculosis in adults, pregnant women, and serodiscordant couples. J Acquir Immune Defic Syndr. 2013;62:e87–97. 23187942 10.1097/QAI.0b013e31827e4992

[25] Evans C , Bennett J , Croston M , Brito‐Ault N , Bruton J . “In reality, it is complex and difficult”: UK nurses’ perspectives on “treatment as prevention” within HIV care. AIDS Care. 2015;27(6):753–7. 25650545 10.1080/09540121.2014.1002826

[26] Buchacz K , Farrior J , Beauchamp G , McKinstry L , Kurth AE , Zingman BS , et al. Changing clinician practices and attitudes regarding the use of antiretroviral therapy for HIV treatment and prevention. J Int Assoc Provid AIDS Care. 2017;16(1):81–90. 27708115 10.1177/2325957416671410 PMC5621922

[27] Krakower DS , Oldenburg CE , Mitty JA , Wilson IB , Kurth AE , Maloney KM , et al. Knowledge, beliefs and practices regarding antiretroviral medications for HIV prevention: results from a survey of healthcare providers in New England. PLoS ONE. 2015;10:e0132398. 26146824 10.1371/journal.pone.0132398 PMC4492498

[28] Krakower DS , Beekmann SE , Polgreen PM , Mayer KH . Diffusion of newer HIV prevention innovations: variable practices of frontline infectious diseases physicians. Clin Infect Dis. 2016;62(1):99–105. 26385993 10.1093/cid/civ736 PMC4678105

[29] UNAIDS . Ending AIDS: Progress towards the 90‐90‐90 targets. [cited 2018 Oct 18]. Available from: http://www.unaids.org/sites/default/files/media_asset/Global_AIDS_update_2017_en.pdf

[30] Rosen S , Maskew M , Fox MP , Nyoni C , Mongwenyana C , Malete G , et al. Initiating antiretroviral therapy for HIV at a patient's first clinic visit: the rapit randomized controlled trial. PLoS Med. 2016;13:e1002015. 27163694 10.1371/journal.pmed.1002015 PMC4862681

[31] Ford N , Migone C , Calmy A , Kerschberger B , Kanters S , Nsanzimana S , et al. Benefits and risks of rapid initiation of antiretroviral therapy. Aids. 2018;32(1):17–23. 29112073 10.1097/QAD.0000000000001671 PMC5732637

[32] Hanna DB , Buchacz K , Gebo KA , Hessol NA , Horberg MA , Jacobson LP , et al. Trends and disparities in antiretroviral therapy initiation and virologic suppression among newly treatment‐eligible HIV‐infected individuals in North America, 2001–2009. Clin Infect Dis. 2013;56(8):1174–82. 23315317 10.1093/cid/cit003 PMC3657490

[33] Mgbere O , Rodriguez‐Barradas M , Vigil KJ , McNeese M , Tabassam F , Barahmani N , et al. Systemic delays in the initiation of antiretroviral therapy for clinically eligible HIV‐infected patients in Houston, Texas: the providers’ report card. J Int Assoc Provid AIDS Care. 2018;17:1–12. 10.1177/2325958218774042 PMC6748492 29745311

[34] Boyd M , Boffito M , Castagna A , Estrada V . Rapid initiation of antiretroviral therapy at HIV diagnosis: definition, process, knowledge gaps. HIV Med. 2019;20(S1):3–11. 10.1111/hiv.12708 30724450

[35] Beer L , Valverde EE , Raiford JL , Weiser J , White BL , Skarbinski J . Clinician perspectives on delaying initiation of antiretroviral therapy for clinically eligible HIV‐infected patients. J Int Assoc Provid AIDS Care. 2015;14(3):245–54. 25394912 10.1177/2325957414557267 PMC4426141

[36] Alemnji G , Chase M , Branch S , Guevara G , Nkengasong J , Albalak R . Improving laboratory efficiency in the caribbean to attain the World Health Organization HIV treat all recommendations. AIDS Res Hum Retroviruses. 2018;34(2):132–9. 28967269 10.1089/AID.2017.0158

[37] Pham MD , Romero L , Parnell B , Anderson DA , Crowe SM , Luchters S . Feasibility of antiretroviral treatment monitoring in the era of decentralized HIV care: a systematic review. AIDS Res Ther. 2017;14(1):3. 28103895 10.1186/s12981-017-0131-5 PMC5248527

[38] Peter T , Zeh C , Katz Z , Elbireer A , Alemayehu B , Vojnov L , et al. Scaling up HIV viral load ‐ lessons from the large‐scale implementation of HIV early infant diagnosis and CD4 testing. J Int AIDS Soc. 2017;20(Suppl 7):e25008. 10.1002/jia2.25008 PMC5978645 29130601

