# Supplementary material for: Implementation of “Treat‐all” at adult HIV care and treatment sites in the Global IeDEA Consortium: results from the Site Assessment Survey
Source: J Int AIDS Soc. 2019 Jul 12;22(7):e25331. doi: 10.1002/jia2.25331 (PMC6625339; doi:10.1002/jia2.25331)
Supplement: Supplementary file 1 — Table S1. Date of national adoption of Treat All, median year, and time‐to‐implementation at IeDEA sites operating under Treat All at the time of the survey. [file JIA2-22-e25331-s001.docx]

**Supplemental Table 1. Date of national adoption of Treat All, median year, and time-to-implementation at IeDEA sites operating under Treat All at the time of the survey**^†^

| **IeDEA region & country** | **Month/Year of national Treat All adoption** | **Number of IeDEA sites reporting year and month of Treat All introduction** | **Median year of site introduction of Treat All (IQR)** | **Median interval (months) from national Treat All adoption to site implementation (IQR)** |
| --- | --- | --- | --- | --- |
| **Asia-Pacific** | |  |  |  |
| Australia ^[1]^ | Aug 2015 | 10 | 2015 [2015, 2016] | -1 (-7, 5) |
| China ^[2, 3]^ | Jun 2016 | 2 | 2015.5 [2015, 2016] | -7 (-11, -3) |
| India ^[4]^ | Apr 2017 | 2 | 2011.5 [2011, 2012] | -63 (-68, -58) |
| Cambodia ^[2, 5]^ | Aug 2016 | 2 | 2017 [2017, 2017] | 5 (5, 5) |
| Japan ^[2]^ | Mar 2016 | 1 | 2016 [2016, 2016] | N/A^§^ |
| South Korea ^[2, 6]^ | Oct 2013 | 1 | 2013 [2013, 2013] | -9 (-9, -9) |
| Malaysia ^[7]^ | Jul 2017 | 2 | 2015.5 [2015, 2016] | -18 (-23, -13) |
| New Zealand ^[8]^ | Jul 2017 | 2 | 2017 [2017, 2017] | -3.1 (-5.6, -0.6) |
| Thailand ^[9]^ | Oct 2014 | 4 | 2014.5 [2014, 2015.5] | 6 (-2, 16) |
| Taiwan ^[10]^ | Jul 2016 | 1 | 2016 [2016, 2016] | -2 (-2, -2) |
| **Caribbean, Central and South America** | | | | |
| Argentina ^[2, 11, 12]^ | Jan 2015 | 1 | 2015 [2015, 2015] | N/A^§^ |
| Brazil ^[13]^ | Dec 2013 | 7 | 2013 [2013, 2015] | 0 (-9, 21) |
| Haiti ^[2, 14]^ | Jul 2016 | 1 | 2016 [2016, 2016] | 0 (0, 0) |
| Mexico ^[15]^ | Nov 2014 | 1 | 2015 [2015, 2015] | 12 (12, 12) |
| **Central Africa** |  |  |  |  |
| Burundi ^[2, 16]^ | 1-Sep-16 | 3 | 2016 [2016, 2017] | 2 (1, 4) |
| Cameroon ^[17]^ | 1-Jun-16 | 3 | 2016 [2016, 2016] | 1 (-1, 4) |
| Rwanda ^[2, 18]^ | 1-Jul-16 | 10 | 2016 [2016, 2016] | 0 (0, 0) |
| **East Africa** | |  |  |  |
| Kenya ^[2, 19]^ | Jul 2016 | 22 | 2016 [2016, 2016] | 1 (-1, 2) |
| Tanzania ^[20]^ | Oct 2017 | 1 | 2016 [2016, 2016] | -17.5 (-19.7, -8.7) |
| Uganda ^[2, 21]^ | Nov 2016 | 11 | 2017 [2017, 2017] | 7 (5.9, 7) |
| **North America** | |  |  |  |
| Canada ^[22]^ | Mar 2012 | 4 | 2015.5 [2012.5, 2016.5] | 46 (7, 55) |
| USA ^[2, 22]^ | Mar 2012 | 18 | 2011.5 [2008, 2015] | -4.5 (-50, 38) |
| **Southern Africa** | |  |  |  |
| Lesotho^‡^  ^[2, 23, 24]^ | Apr 2016 | 1 | 2016 [2016, 2016] | 3 (3, 3) |
| Malawi ^[2, 25]^ | May 2016 | 22 | 2016 [2016, 2016] | 2 (2, 2) |
| South Africa ^[2, 26]^ | Sep 2016 | 8 | 2016 [2016, 2016] | 0 (0, 0.5) |
| Zambia^‡^ ^[27]^ | Dec 2016 | 1 | 2016 [2016, 2016] | 0 (0, 0) |
| Zimbabwe^‡^ ^[2, 28, 29]^ | Dec 2016 | 2 | 2017 [2017, 2017] | 1 (1, 1) |

^†^ Countries where Treat All was adopted in national treatment guidelines after the IeDEA survey was completed include: Chile, Democratic Republic of Congo, Republic of the Congo, and Vietnam.

^‡^ One of the sites surveyed represents a cohort of clinics that implement a uniform HIV care and treatment program.

^§^ Only year of Treat All introduction reported.

**References**

1. Australasian Society for HIV VHaSHMA. Antiretroviral Guidelines. 2015; <http://arv.ashm.org.au/clinical-guidance>. Accessed August 11, 2018, 2018.

2. International Association of Providers of AIDS Care (IAPAC). Global HIV Policy Watch. <http://www.hivpolicywatch.org/index.html>. Accessed August 10, 2018.

3. National Health Commission of the People's Republic of China. Antiviral therapy for HIV/AIDS offered free. June 20, 2016; <http://en.nhfpc.gov.cn/2016-06/20/c_70012.htm>. Accessed August 12, 2018.

4. Ministry of Health and Family Welfare (Government of India). Test and Treat Policy for HIV Patients. August 4, 2017; <http://pib.nic.in/newsite/PrintRelease.aspx?relid=169598>. Accessed August 13, 2018.

5. National Centre for HIV/AIDS Dermatology and STD (NCHADS). National HIV Clinical Management Guidelines for Adults and Adolescents. 2016; <https://www.nchads.org/Guideline/HIV%20Magt%20guidelines_English%20Version-3-8-16%20NCHADS.pdf>. Accessed August 13, 2018.

6. The Korean Society for AIDS. The 2013 Clinical Guidelines for the Diagnosis and Treatment of HIV/AIDS in HIV-Infected Koreans. *Infection & Chemotherapy.* 2013;45(4):455-461.

7. Malaysian Society for HIV Medicine (MASHM). Malaysian Consensus Guidelines on Antiretroviral Therapy. 2017; <https://docs.wixstatic.com/ugd/70e62f_f4a5a43502d846d7a8b3cdf43d14f3ea.pdf>, <https://www.mashm.net/resources>. Accessed August 13, 2018, 2018.

8. Government of New Zealand. Decision relating to widening funding criteria for antiretroviral agents for the treatment of HIV. June 12, 2017; <https://www.pharmac.govt.nz/news/notification-2017-06-12-antiretroviral/>. Accessed August 13, 2018.

9. Ministry of Public Health (Government of Thailand). AIDS Prevention Measures. October 1, 2014; <http://pr.moph.go.th/iprg/include/admin_hotnew/show_hotnew.php?idHot_new=68231>. Accessed August 13, 2018.

10. Personal communication. Local expert, Dr Wong Wing-Wai. July 30, 2018.

11. Cesar C, Blugerman G, Valiente JA, et al. The HIV care cascade in Buenos Aires, Argentina: results in a tertiary referral hospital. *Revista panamericana de salud publica = Pan American journal of public health.* 2016;40(6):448-454.

12. Grinsztejn B. HIV Prevention in Latin America - setting the stage. 2017; <https://www.iasociety.org/Web/WebContent/File/EduFundBrazil_13Presentation_BeatrizGrinsztejn.pdf>.

13. Ministério da Saúde. Ministério da Saúde estende tratamento para todos com HIV. December 1, 2013; <http://www.aids.gov.br/pt-br/noticias/ministerio-da-saude-estende-tratamento-para-todos-com-hiv>. Accessed August 12, 2018.

14. U.S. President’s Emergency Plan for AIDS Relief (PEPFAR). PEPFAR Haiti Country Operational Plan (COP) 2017, Strategic Direction Summary. <https://www.pepfar.gov/documents/organization/272014.pdf>. Accessed August 12, 2018.

15. Centro Nacional para la Prevención y el Control del VIH y Sida (Censida). Guía de manejo antirretroviral de las personas con VIH. 2014; <http://www.censida.salud.gob.mx/descargas/principal/Guia_ARV_2014V8.pdf>. Accessed August 12, 2018.

16. Ministère de la Santé Publique et de la Lutte contre le SIDA (Republique du Burundi). Directives nationales d'utilisation des antiretroviraux pour la prevention et le traitement du VIH. September 2016; <https://aidsfree.usaid.gov/sites/default/files/burundi_art_2016.pdf>. Accessed August 13, 2018.

17. U.S. President's Emergency Plan for AIDS Relief (PEPFAR). Cameroon Country Operational Plan (COP) 2017, Strategic Direction Summary. April 17, 2017; <https://www.pepfar.gov/documents/organization/272006.pdf>. Accessed August 11, 2018.

18. U.S. President’s Emergency Plan for AIDS Relief (PEPFAR). Rwanda Country Operational Plan (COP) 2016, Strategic Direction Summary. <https://www.pepfar.gov/documents/organization/257633.pdf>. Accessed August 11, 2018.

19. Ministry of Health (Government of Kenya). Ministry of Health and National AIDS and STI Control Program (NASCOP) Press Release. July 15, 2017; <http://www.health.go.ke/wp-content/uploads/2016/07/Press-release-HISTORIC-MOMENT-AS-KENYA-LAUNCHES-REVOLUTIONARY-HIV-TREATMENT.pdf>. Accessed August 13, 2018.

20. U.S. President’s Emergency Plan for AIDS Relief (PEPFAR). Tanzania Country Operational Plan (COP) 2017, Strategic Direction Summary. March 2, 2017; <https://tz.usembassy.gov/wp-content/uploads/sites/258/2017/07/TZ-COP-2017-SDS-FINAL-Update-29June2017.pdf>. Accessed August 12, 2018.

21. Ministry of Health (Republic of Uganda). Consolidated Guidelines for Prevention and Treatment of HIV in Uganda. November 18, 2018; <http://library.health.go.ug/publications/service-delivery-diseases-control-prevention-communicable-diseases/hivaids/consolidated>. Accessed August 13, 2018.

22. Department of Health and Human Services (USA). Guidelines for the use of antiretroviral agents in HIV-1-infected adults and adolescents. March 27, 2012; <https://aidsinfo.nih.gov/contentfiles/AdultandAdolescentGL003093.pdf>. Accessed August 12, 2018.

23. U.S. President’s Emergency Plan for AIDS Relief (PEPFAR). Lesotho Country Operational Plan (COP) 2016, Strategic Direction Summary. May 20, 2016; <https://www.pepfar.gov/documents/organization/257640.pdf>. Accessed August 13, 2018

24. U.S. Embassy in Lesotho. Remarks By PEPFAR Lesotho Coordinator Reuben Haylett at the Launch of the Revised National ART Policy Guidelines: Test and Treat. April 14, 2016; <https://ls.usembassy.gov/remarks-pepfar-lesotho-coordinator-reuben-haylett-launch-revised-national-art-policy-guidelines-test-treat/>. Accessed August 13, 2018.

25. Malawi Guidelines for Clinical Management of HIV in Children and Adults. May 2016; <https://aidsfree.usaid.gov/sites/default/files/malawi_art_2016.pdf>. Accessed August 13, 2018.

26. U.S. President’s Emergency Plan for AIDS Relief (PEPFAR). Country Operational Plan (COP) 2017 Approval Meeting Outbrief. 2017; <https://za.usembassy.gov/wp-content/uploads/sites/19/2017/07/COP17-Approval-Meeting-Outbrief.pdf?_ga=2.55839339.1301664358.1534022522-1838988652.1534022522>. Accessed August 13, 2018.

27. U.S. Embassy in Zambia. Hands Up for HIV Treatment: Zambians to Save Lives through Adoption of Test & Start for HIV Treatment. December 20, 2016; <https://zm.usembassy.gov/test-and-start/>. Accessed August 13, 2018.

28. Ministry of Health and Child Care (MOHCC). Guidelines for Antiretroviral Therapy for the Prevention and Treatment of HIV in Zimbabwe. December 2016; <https://aidsfree.usaid.gov/sites/default/files/zw_arv_therapy_prevention.pdf>. Accessed August 13, 2018.

29. Ministry of Health and Child Care (MOHCC). Operational and Service Delivery Manual for the Prevention, Care and Treatment of HIV in Zimbabwe. February 2017; <http://ophid.org/treat-all-toolkit/MOHCC%20Guidelines/MOHCC%20Zimbabwe%20OSDM%202017.pdf>. Accessed August 13, 2018.
